# *Providencia stuartii* form biofilms and floating communities of cells that display high resistance to environmental insults

**DOI:** 10.1371/journal.pone.0174213

**Published:** 2017-03-23

**Authors:** Mariam El Khatib, Que-Tien Tran, Chady Nasrallah, Julie Lopes, Jean-Michel Bolla, Michel Vivaudou, Jean-Marie Pagès, Jacques-Philippe Colletier

**Affiliations:** 1 Institut de Biologie Structurale, Université Grenoble Alpes – Commissariat à l’Énergie Atomique – Centre National de la Recherche Scientifique, Grenoble, France; 2 School of Biophysics, Jacobs University of Bremen, Bremen, Germany; 3 Équipe Transporteurs Membranaires, Chimiorésistance et Drug-Design, Université Aix-Marseille – Institut de Recherche Biomédicale des Armées, Marseille, France; National Institute of Technology Rourkela, INDIA

## Abstract

Biofilms are organized communities of bacterial cells that are responsible for the majority of human chronic bacterial infections. *Providencia stuartii* is a Gram-negative biofilm-forming bacterium involved in high incidence of urinary tract infections in catheterized patients. Yet, the structuration of these biofilms, and their resistance to environmental insults remain poorly understood. Here, we report on planktonic cell growth and biofilm formation by *P*. *stuartii*, in conditions that mimic its most common pathophysiological habitat in humans, *i*.*e*. the urinary tract. We observed that, in the planktonic state, *P*. *stuartii* forms floating communities of cells, prior to attachment to a surface and subsequent adoption of the biofilm phenotype. *P*. *stuartii* planktonic and biofilm cells are remarkably resistant to calcium, magnesium and to high concentrations of urea, and show the ability to grow over a wide range of pHs. Experiments conducted on a *P*. *stuartii* strain knocked-out for the Omp-Pst2 porin sheds light on the role it plays in the early stages of growth, as well as in the adaptation to high concentration of urea and to varying pH.

## Introduction

Bacteria are known to live as organized community of cells termed biofilms. In humans, these supra-cellular structures are responsible for the majority of chronic bacterial infections [1,2]. Prominent examples of biofilm-related infections include catheter-associated infections, the leading cause of secondary nosocomial bacteremia (20%) [3], and cystic fibrosis [4], a genetic disorder that favours the colonisation of aerial tissues by *P*. *aeruginosa*. The chronic nature of biofilm-related infections originates from their increased resistance to the immune system and antibiotherapy. The current model for biofilm formation includes five different stages [5], *viz*. i/ the initial attachment of cells on a biotic or abiotic surface; ii/ the formation of a monolayer of cells; iii/ the migration of cells into a multi-layered colony; iv/ the synthesis of an extracellular matrix around the cells; and v/ the maturation of the biofilm into a characteristic 3D ensemble, composed of cells flapping in a self-produced polymeric matrix [5]. A sixth stage would be the release and dispersion of biofilm cells to colonize other niches. Depending on species, the biofilm polymeric matrix may be composed of extra-cellular polysaccharides [1], amyloid fibers [6] and DNA [7]. The versatile and adaptable nature of the matrix allows bacteria to attach on, and thus colonize, a range of disparate (biotic or abiotic) surfaces. It also affects antibiotic efficiency through a variety of mechanisms, including reduced diffusion of drugs within the biofilm, masking or alteration of drug targets by the biofilm environment, or the adoption by some cells of a dormant—and therefore less drug-susceptible—phenotype [8,9].

*Providencia stuartii* is an opportunistic biofilm-forming pathogen from the *Enterobacteriacae* family [10] that is ubiquitous in the environment [11]. A recent study reported an incidence rate of 4 per 100,000 hospital admissions, suggesting a low rate of prevalence in the general population [11]. *P*. *stuartii* is yet responsible for ≈ 9% of urinary tract infections, in patients undergoing long-term catheterization [11–15]. These patients are often nursing home (NH) or intensive care unit (ICU) residents; hence this contingent is bound to increase with aging of the population. The in-hospital mortality rate of *P*. *stuartii* infections is around 30% [11], in part due to its high intrinsic multidrug resistance (MDR) phenotype conferred by the presence of an inducible chromosomal AmpC [16]. This MDR phenotype can be further exaggerated in clinical isolates, a majority of which were shown to feature plasmid-encoded extended-spectrum β-lactamases (ESBLs) [17]. More recently, clinical isolates presenting carbapenemase activities were isolated in Afghanistan [18] and Portugal [19]. *P*. *stuartii* is adept at biofilm dispersion, explaining that infections sometimes migrate from the urinary tract to other organs, causing endocardisis [20], pericarditis [21], peritonitis [22] or meningitis [23]. These facts, together with the now established ability of *P*. *stuartii* to disseminate amidst patients in hospital settings [24,25], explain the growing concern among health professionals [11]. As yet, however, studies remain scarce on *P*. *stuartii* and on the nature and resistance of its biofilms to environmental cues [26–28]. More investigations are needed to characterize how *P*. *stuartii* biofilms form, and what their specifics are in terms of extracellular matrix composition, cell sub-types and behaviour, and mechanisms of adherence-to and detachment-from surfaces or other cells. Such information is crucial to eventually prevent or manage chronic infections by *P*. *stuartii*, and the high toll they take on NH and ICU residents [11,29].

In urine, the metabolite urea is found at a concentration of 150 mM, at which it displays a strong anti-microbial effect. Thus, bacteria that colonize the urinary tract must find means to evade this stress. One of these is to feature (or acquire) a urease activity, that will hydrolyze urea into two ammoniums and an carbonate [30]. Calcium and magnesium are generally found at normal serum concentration (2 and 2.5 mM) but may reach higher concentrations in pathological conditions, e.g. in patients presenting bladder stones whose formation correlates with a 2-fold increase in the calcium-concentration/osmolarity ratio of urine [31,32]. The pH of urine is usually acidic but may vary from 6 to 8 depending on diet or pre-conditions. For example, infection by *P*. *mirabilis* is known to raise urine pH above 8, due to its strong urease activity that degrades urea into carbon monoxide and ammonia [29]. Some clinical isolates of *P*. *stuartii* feature a plasmid-encoded urease activity, but this activity is generally too weak to induce alkalinisation of urine [33]. Therefore alternative mechanisms, which allow *P*. *stuartii* cells to evade detrimental effects of urea, must exist. One of these is co-infection with species that have a strong urease activity such as *P*. *mirabilis*, whose presence was shown to increase *P*. *stuartii* colonization and bacteremia incidence [34]. Another efficient mechanism could be the limited diffusion of urea across the extracellular matrix of *P*. *stuartii* biofilms, which would result in reducing the effective concentration of urea in cells, thence preserving these. Access to the periplasm is mainly controlled by general-diffusion porins, which are water-filled channels sprinkling the outer-membrane thence allowing passive diffusion of nutrients and ions into the periplasm. Porins are the most abundantly expressed outer-membrane (OM) proteins (up to 100,000 copies/cell), with a single porin often accounting for up to 70% of the OM proteinaceous content [35]. Current interest in porins mostly stems from their involvement in antibiotic uptake [36] and in the emergence of antibiotic resistance [37]. But as the first door opened toward the exterior, they also play a number of additional roles in bacterial survival, homeostasis and pathogenesis, adhesion to surfaces and host cells [38], and sometimes penetration into these [39]. Porins are therefore good candidates for playing a role in limiting excessive urea accumulation in the periplasm.

The genome of *P*. *stuartii* features two porins, Omp-Pst1 and Omp-Pst2 [40]. When grown in rich medium (*per se*, Luria-Bertani or LB), *P*. *stuartii* predominantly expresses Omp-Pst1, and it was proposed that Omp-Pst1 is the major porin of the bacterium [40]. Electrophysiology measurements revealed that Omp-Pst2 is highly cation-selective and prone to voltage-gating (critical voltage Vc = 20–90 mV), whereas Omp-Pst1 channel gates normally (Vc > 199 mV), is mildly anion selective and comparatively more permissive to β-lactam antibiotics [40,41]. MD simulations suggested that Omp-Pst2 atypical voltage-gating behaviour is asymmetric and triggered by the influx of cations from the extracellular to the periplasmic side of the porin. Efflux of cations, on the other hand, would be facilitated, suggesting a potential role for this porin in the regulation of charge distribution across the OM [42].

Here, we report on *P*. *stuartii* growth and biofilm formation under environmental conditions that mimic its most common habitat in humans, *i*.*e*. the urinary tract. We used the methodology of Mishra *et al*. [43] to characterize the effect of pH, urea, calcium and magnesium on biofilm genesis, attachment and consolidation. We found that *P*. *stuartii* growth is independent on pH in the viability range (pH 6 to 9), yet biofilm genesis and attachment onto the surface are favoured at pH ≥ 8. We observed that *P*. *stuartii* biofilms survive in high concentrations of urea (up to 500 mM), calcium and magnesium (up to 50 mM), and that these environmental stresses trigger the consolidation of *P*. *stuartii* biofilms. Magnesium and calcium both inhibit the attachment of new cells onto surfaces in a dose-dependent manner, but magnesium activates biofilm genesis. Epifluorescence micrographs were taken at various stages of growth, of cells both in the planktonic (floating cells) and in the biofilm state (adherent cells). Most unexpectedly, we observed that planktonic *P*. *stuartii* cells exhibit a highly social behaviour, whereby cell-to-cell contact occurs prior to attachment of cells onto the surface, resulting in floating communities of cells that precede—and later coexist—with surface-attached biofilms. This observation suggested cell-to-cell contact as the primary mechanism by which *P*. *stuartii* cells form a community, and prompted us to examine whether or not porins—as the main proteinaceous component of the outer membrane—are involved. A knock-out strain for Omp-Pst2, *P*. *stuartii ΔP2*, was obtained (*P*. *stuartii ATCC 29914 ΔompPst2*::*Cm*) that formed more biofilms (+70%) but displayed retarded growth, higher sensitivity to urea and cations, and a clear dependence of the lag-time on pH. Results suggest that Omp-Pst2 is an important actor in the early stages of *P*. *stuartii* growth and in adaptation to alkalinity.

## Materials and methods

### Strains and materials

*Escherichia coli* BL21 DE3 strain was used as a negative control strain and the wild type *Providencia stuartii ATCC 29914* strain was obtained from the Pasteur Institute (Paris, France). All fluorescent dyes were from Thermo Fischer Scientific. Unless specified otherwise, chemicals were from Sigma-Aldrich. Polystyrene-bottom 96-well plates were from Greiner.

### Generation of the *omp-pst2* knock out strain, *P*. *stuartii* ΔP2

The disruption of the *omp-Pst2* gene in *P*. *stuartii* ATCC 29914 was carried out by adapting the protocol described by Datsenko and Wanner [44], based on the use of phage lambda Red recombinase [44]. The PCR primers were OmpPst2_XbaI 5’- GTG TCT AGA CAC TTA GTT AGT AAA TGG C -3’ (forward) and OmpPst2_BamHI 5’- GTT GGA TCC GGA TAA TTG CGT ATG ATG G -3’ (reverse). The *ompPst2* PCR amplicon was cloned into pGem-T-Easy vector and the construct was transferred by electroporation into *E*. *coli* DH5α for plasmid maintenance and amplification. The plasmid was digested with HindIII enzyme and the subsequent protruding ends were filled in by Klenow enzyme. The construct was then ligated to chloramphenicol-resistance (Cm) cassette making *omp-Pst2*::*Cm* knockout construct. pCAM-MSC suicide vector for *Enterobacteriaceae* was then used to bring the *omp-Pst2*::*Cm* into *E*. *coli S17-1λpir*. Biparental mating with *P*. *stuartii* was then carried out by homology conjugation. Selection of *P*. *stuartii* ATCC 29914 *omp-Pst2*::*Cm* mutants was performed in the presence of chloramphenicol at 33 μg/mL concentration. The resulting genetic modification of *P*. *stuartii* ATCC 29914 *omp-Pst2*::*Cm* was finally confirmed by both colony PCR and sequencing, using primers flanking the *omp-Pst2* gene in the *P*. *stuartii* genome.

### Bacterial growth studies

*E*. *coli* and *P*. *stuartii* bacteria were grown in Luria-Bertani (LB) growth medium without antibiotics. LB medium was supplemented with 33 μg/ml chloramphenicol for the growth of the *P*. *stuartii ΔP2* bacteria. Control experiments showed that chloramphenicol at this concentration has no effect on cell-growth and biofilm formation by *P*. *stuartii*-ΔP2 (data not shown). For each experiment, a single bacterial colony was inoculated in standard LB or pH-specific LB medium for 2 h, yielding cells in their lag phase. These were then distributed into a 96-wells plates supplemented with 0–1 M urea, 0–50 mM Ca^2+^ or 0–50 mM Mg^2+^, and incubated at 37°C and under 100 rpm agitation overnight, to form biofilms. Bacterial growth was monitored in terms of absorbance at 600 nm for 24 h (10 minutes interval between time points) using a Biotek Synergy H4 microplate reader (Winooski, VT, USA)

### Environmental stresses impact on biofilm genesis and attachment onto surface

For each experiment, a single bacterial colony was used to inoculate 25 mL of standard LB medium (37°C). A large flask was used, that was maintained under constant shaking at 200 rpm on the rotatory platform, thence preventing bacterial attachment onto the surface and subsequent formation of adherent biofilms. After 2 hours, aliquots of this culture (cells in the lag phase) were transferred in a 96-well plate (see below), serving to study the effect of environmental stresses on biofilm genesis. After 24 h, another set of aliquots was transferred in the 96-well plate, with view to study the effect of environmental stresses on the attachment of cells onto the well surfaces (see below). Cells were confronted with environmental insults after transfer into the 96-well plate. Briefly, above-mentioned aliquots were distributed into wells containing 150 μL LB medium buffered at increasing pH (4 to 9) or supplemented with increasing concentrations of urea (0–1 M), Ca^2+^ or Mg^2+^ (0–0.05 M). Cells were then incubated at 37°C under constant shaking at 100 rpm. Biofilms were revealed after 24-hours incubation in the 96-well plate.

### Environmental stresses impact on established biofilms

For each experiment, a single bacterial colony was used to inoculate 25 mL of standard LB medium (37°C). A large flask was used, that was maintained under constant shaking at 200 rpm on the rotatory platform, thence preventing bacterial attachment onto the surface and subsequent formation of adherent biofilms. After 2 hours, aliquots of this culture (cells in the lag phase) were transferred in a 96-well plate and incubated for 24 hours at 37°C under constant shaking at 100 rpm, resulting in the formation of biofilms at the bottom of the wells. The medium was then removed to discard planktonic bacteria and replaced by fresh LB medium buffered at increasing pH (4 to 9) or supplemented with urea (0–1 M), Ca^2+^ or Mg^2+^ (0–0.05 M). The plate was then incubated at 37°C and 100 rpm overnight. Biofilms were revealed after an additional 24-hours incubation in this plate.

### Imaging

Epifluorescence micrographs were taken on an IX81 Olympus inverted microscope; samples were magnified through 20 or 60X objectives (Plan APON60XO, Olympus). SYTO^®^ 9 Green fluorescent nucleic acid stain and propidium iodide solution were used at 5 and 20 μM concentration to stain live and dead cells respectively. Bacterial membranes were stained by the fluorescent dye FM1-43FX at 5 μg/mL. Bacterial biofilms were grown in a 96-wells plates as described above. For the imaging of planktonic bacteria, 10 μL of the culture were spread on LB-Gelzan^™^ cover slides prepared as previously described [45] and imaged directly afterwards. For biofilm imaging, wells were washed twice with phosphate-buffered saline (PBS) to remove all planktonic bacteria; the remaining adherent bacteria forming the biofilm were then imaged.

### Biofilm quantification

Well plates incubated overnight were washed extensively with PBS to remove all planktonic bacteria. Adherent biofilms were then stained with PrestoBlue^®^ cell viability reagent. For each plate, fluorescence emission was measured at 590 nm, upon excitation at 560 nm. All experiments were performed at least in triplicate and biofilm formation was quantified with respect to *P*. *stuartii* cells grown in the absence of environmental stresses.

## Results

### *P*. *stuartii* form floating communities of cells prior to adherent biofilm

**Fig 1:**
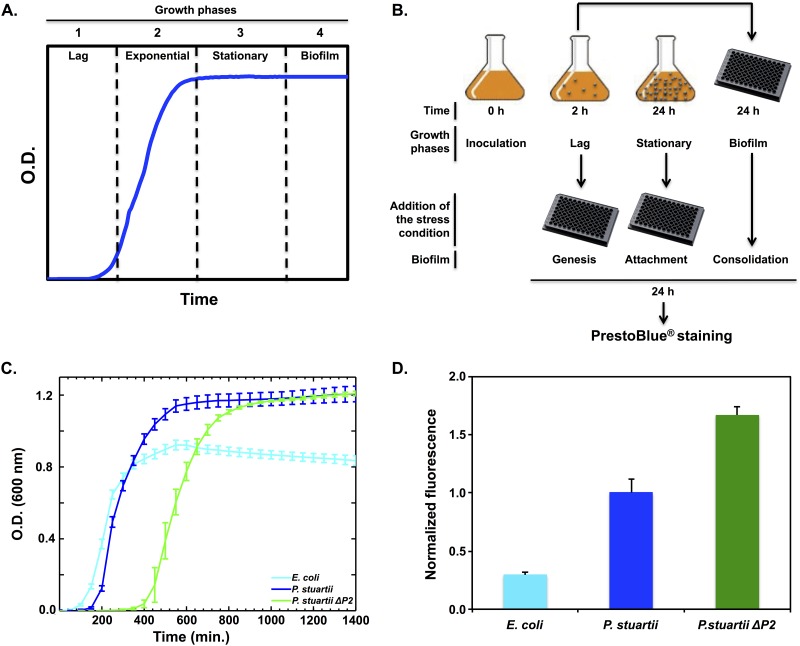
Planktonic bacterial growth and biofilm formation. (A) Bacterial growth can be divided into 4 main phases: (1) the lag phase, (2) the exponential phase, (3) the stationary phase and (4) the biofilm establishment phase. (B) Growth curves of *E*. *coli* BL21, *P*. *stuartii* and *P*. *stuartii-*ΔP2. (C) Experimental protocol designed to challenge the effect of various environmental stresses on the different stages of biofilm formation (genesis, attachment, consolidation) by *P*. *stuartii*. (D) Biofilm quantification after 24h of growth in 96-wells plates, as revealed by of adherent cells using PrestoBlue. Data are normalized with respect to measurements performed on *P*. *stuartii*.

In order to characterize *P*. *stuartii* growth and its ability to form biofilms, we compared it to one of the most common *E*. *coli* strains used in laboratories, BL21 DE3. This *E*. *coli* strain is known not to form biofilms [46] and was therefore used as a negative control. When cultivated in LB medium, both *P*. *stuartii* and *E*. *coli* show a typical growth curve that can be divided into three phases: (1) a lag phase, (2) an exponential phase, and (3) a stationary phase (Fig 1A). *E*. *coli* and *P*. *stuartii* have similar lag phase duration and the same growth rate in the beginning of the exponential phase (Fig 1B). After five hours, however, *E*. *coli* cells decline whereas *P*. *stuartii* cells continue to exhibit a positive rate of growth.

**Fig 2:**
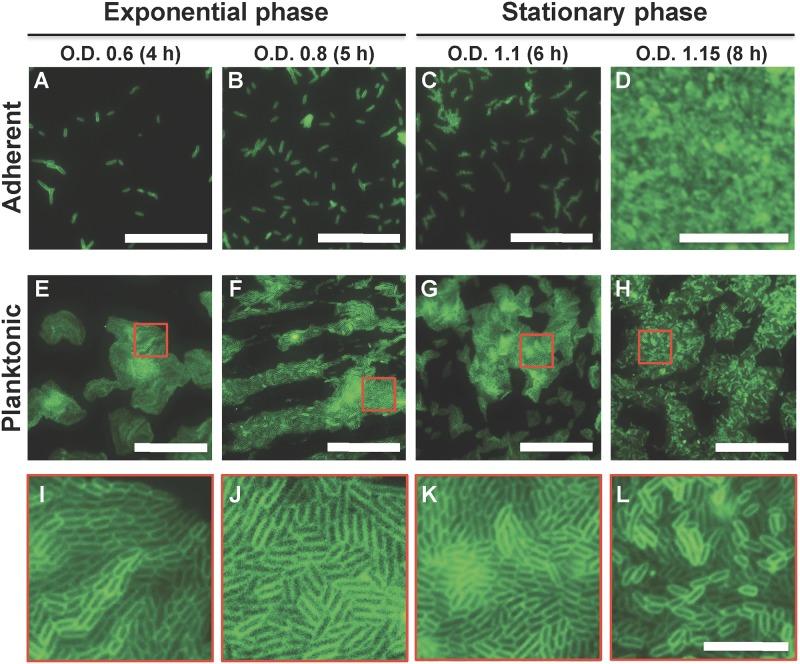
*P*. *stuartii* form floating-communities of planktonic-cells prior to adherent biofilms. (A-D) Micrographs of bacteria that remain bound to the well surface, after discard of planktonic cells by PBS washes. (E-H) Micrographs of planktonic bacteria. Cells were pipetted from the LB medium, spreaded on LB-Gelzan and imaged immediatly. (I-L) Close-up views of the red-delineated regions in panels E-H showing close contact between the membrane of adjacent cells. Bacterial membranes were stained using FM1-43FX. Scale bars: 50 μm (A-H) and 10 μm (I-L).

Micrographic examination of attached *P*. *stuartii* cells reveals that no biofilm forms before the stationary phase (Fig 2A–2C), in agreement with the idea that bacteria form biofilms when faced with an environmental stress. After five hours of growth, the well is covered with a large biofilm, presumably as a result of starvation (Fig 2D). Examination of planktonic cells at the exponential and stationary phases reveals that floating communities of cells form by cell-to-cell contact in solution (Fig 2E–2G and S1 Movie), prior to the attachment of cells on the surface (Fig 2A–2C)–that is, prior to the formation of canonical biofilms. After five hours of growth, these floating communities of cells are multi-layered and coexist with surface-attached biofilms (Fig 2D–2H).

**Fig 3:**
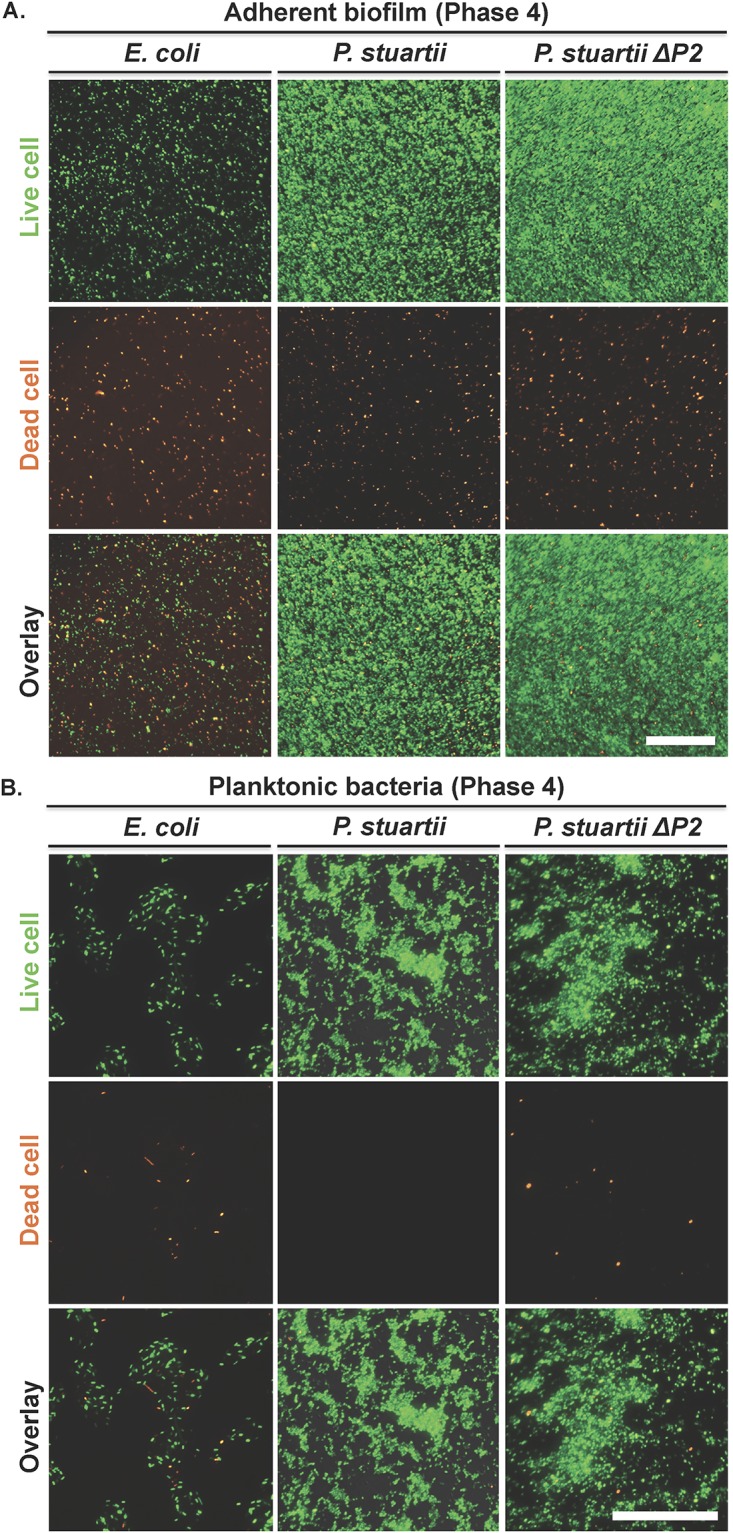
*P*. *stuartii* is adept at forming large biofilms mainly composed of living cells. (A) Micrographs of bacteria adherent to the wells surface after discard of planktonic cells by PBS washes. (B) Micrographs of planktonic bacteria. Cells were pipetted from the LB medium, spreaded on LB-Gelzan^®^ and imaged immediatly. Live and dead cells were stained with SYTO9 Green and propidium iodide, respectively. Scale bars are 100 and 50 μm in panels (A) and (B), respectively.

The viable adherent biomass formed by *P*. *stuartii* was 3-fold greater than that formed by *E*. *coli* (Fig 1C), with virtually all cells being alive in *P*. *stuartii* biofilms (Fig 3A). In contrast, *E*. *coli* adherent biomass amounted to only few dispersed cells, roughly the half of which were dead (Fig 3A). Phenotypic differences between *P*. *stuartii* and *E*. *coli* cells were also clear at the planktonic level, where the dispersion of *E*. *coli* cells contrasted with the highly gregarious behaviour of *P*. *stuartii* floating communities (Fig 3B).

We examined whether Omp-Pst2 could play a role in biofilm formation by repeating these experiments on the *P*. *stuartii ATCC 29914 ΔompPst2*::*Cm* strain (*P*. *stuartii ΔP2*). The growth rate of *P*. *stuartii ΔP2* in the exponential and stationary phases was identical to that of the wild-type strain, showing a rapid adaptation to Omp-Pst2 depletion (Fig 1B). However, cells devoid of Omp-Pst2 displayed retarded growth (+ 60% increase in lag time; Fig 1B) and a 70% increase in biofilm formation (Fig 1C). *P*. *stuartii ΔP2* biofilms were comparatively denser, but the lack of Omp-Pst2 did not perturb the microscopic appearance of *P*. *stuartii* cells—in either planktonic (Fig 3A) or biofilm (Fig 3B) states. Increased biofilm formation by *P*. *stuartii ΔP2* could underlie a pathway undertaken by this K.O. strain to overcome the stress induced by the lack of Omp-Pst2 in the early stages of growth.

### *P*. *stuartii* is resistant to urea and forms biofilm over a large range of pH

**Fig 4:**
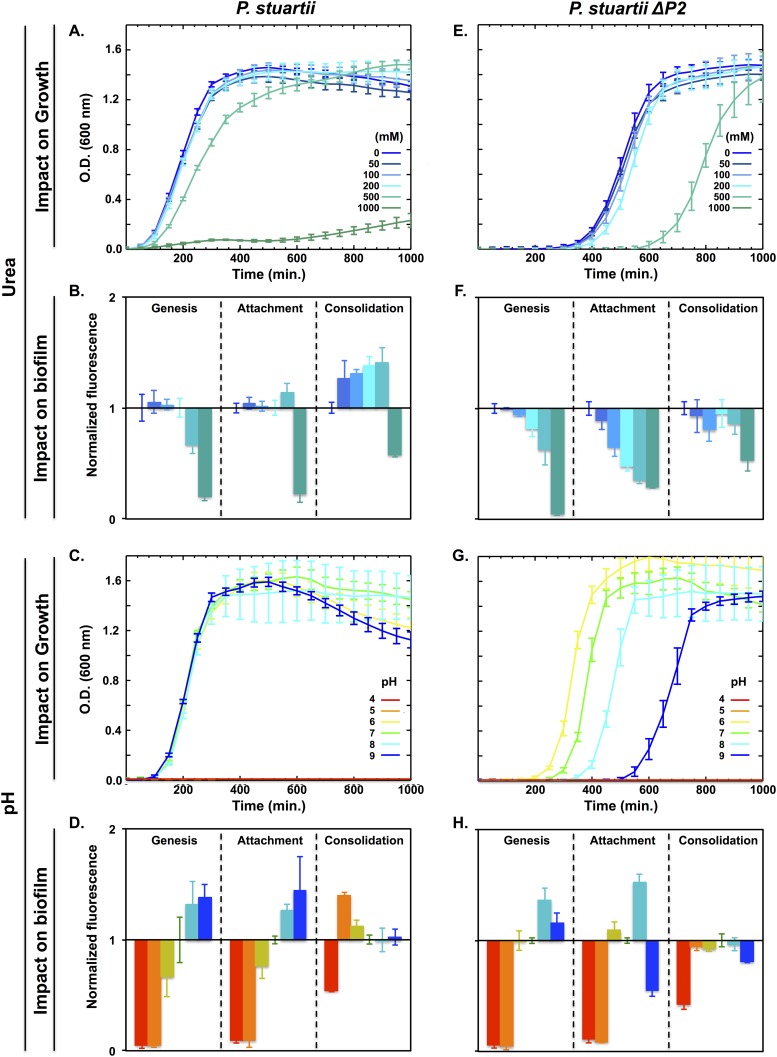
Resistance of *P*. *stuartii* and *P*. *stuartii ΔP2* cells to high concentrations of urea and to pH variations. Effect of urea (A and E) and pH (C and G) on bacterial growth, as judged from the O.D. of cultures at 600 nm. The impact on biofilm genesis, attachment and consolidation was evaluated by adding urea (B and F) or changing the pH (D and H) during the lag, stationary and preformed biofilm phases, respectively. Biofilm formation was quantified 24 h later, by PrestoBlue staining. Fluorescence values were normalized with respect to the control wells containing no urea and buffered at pH7.

Inasmuch as urea is the principal component of urine, we set to determine the effect of this metabolite on *P*. *stuartii* planktonic cell growth as well as on biofilm genesis, attachment and consolidation. We used to this end the methodology recently introduced by Mishra *et al*. [43]. Briefly, cells were submitted to increasing urea concentration at various phases of their growth (lag, stationary and biofilm phases), grown overnight (ON) under this environmental pressure, and the viable biofilm mass was then quantified using Presto Blue (Fig 1D). We found that *P*. *stuartii* planktonic cells sustain normal growth up to 500 mM urea but display an 85% decrease in growth at 1 M urea (Fig 4A). Exposure of cells to urea in the lag and stationary phases reveals that biofilm genesis and attachment to surfaces are unaffected by concentrations of urea up to 200 and 500 mM, respectively (Fig 4B and S1 Fig). Exposure to urea furthermore consolidates pre-formed *P*. *stuartii* biofilms, in the viability range (0–500 mM) (Fig 4B and S2 Fig). Clearly, ATCC 29914 is a urease-positive *P*. *stuartii* strain, sustaining urea concentrations ≥ 4-fold higher than that encountered in its human habitat (150 mM). The urease activity of *P*. *stuartii* is cytoplasmic and produces 2 ammoniums and 1 carbonate per hydrolysed molecule of urea [30].

We then investigated how *P*. *stuartii* responds to changes in pH. Using the approach summarized above—and further detailed in the Methods section—we found that *P*. *stuartii* is unable to grow at pH 4 and 5, but viable between pH 6 and 9 (Fig 4C). Within this range, neither planktonic cells (Fig 4C) nor cells from preformed biofilms are affected by pH variations, although alkalinity favours biofilm genesis and attachment of cells onto the surface (Fig 4D and S1 Fig). While partially destroyed at pH 4, preformed *P*. *stuartii* biofilms consolidate at pH 5 (Fig 4D and S2 Fig) underlining their resistance to extreme environmental conditions.

### Omp-Pst2 is involved in urea uptake and in the regulation of pH in the periplasm

We examined the impact of urea on planktonic growth and biofilm formation by the ΔOmp-Pst2 strain of *P*. *stuartii*. *P*. *stuartii ΔP2* showed normal growth in urea up to 200 mM, but an increased lag time at 500 mM. At 1M, the strain could not grow, suggesting that Omp-Pst2 participates in alleviating urea toxicity in parental cells (Fig 4E). The fitness loss induced by lack of Omp-Pst2 was also visible at the biofilm level, with biofilm genesis, attachment and consolidation all being negatively impacted by urea in a dose-dependent manner (Fig 4F and S1 and S2 Figs).

We also investigated the sensitivity of *P*. *stuartii* ΔP2 to variations in pH. At the planktonic level, we observed a clear dependency on pH of the lag time of *P*. *stuartii ΔP2* growth. The K.O. strain remains unable to grow at pH 4 and 5, but displays a faster and more productive planktonic growth at pH 6 than at pH 9 (Fig 4G). In strong contrast, the growth of the parental strain is equally favoured from pH 6 to 9 (Fig 4C). At the biofilm level, *P*. *stuartii* ΔP2 cells show reduced biofilm genesis and attachment to the surface at pH 9, resulting in a shift of the optimal pH for biofilm formation from pH 9 to 8 (Fig 4H and S1 Fig). Absence of Omp-Pst2 also lowers biofilm consolidation at pH5, as compared to the parental strain (Fig 4D and 4H and S2 Fig). Altogether, these results indicate a role for Omp-Pst2 in the regulation of periplasmic pH.

### *P*. *stuartii* biofilms benefit from the presence of Ca^2+^ and Mg^2+^

**Fig 5:**
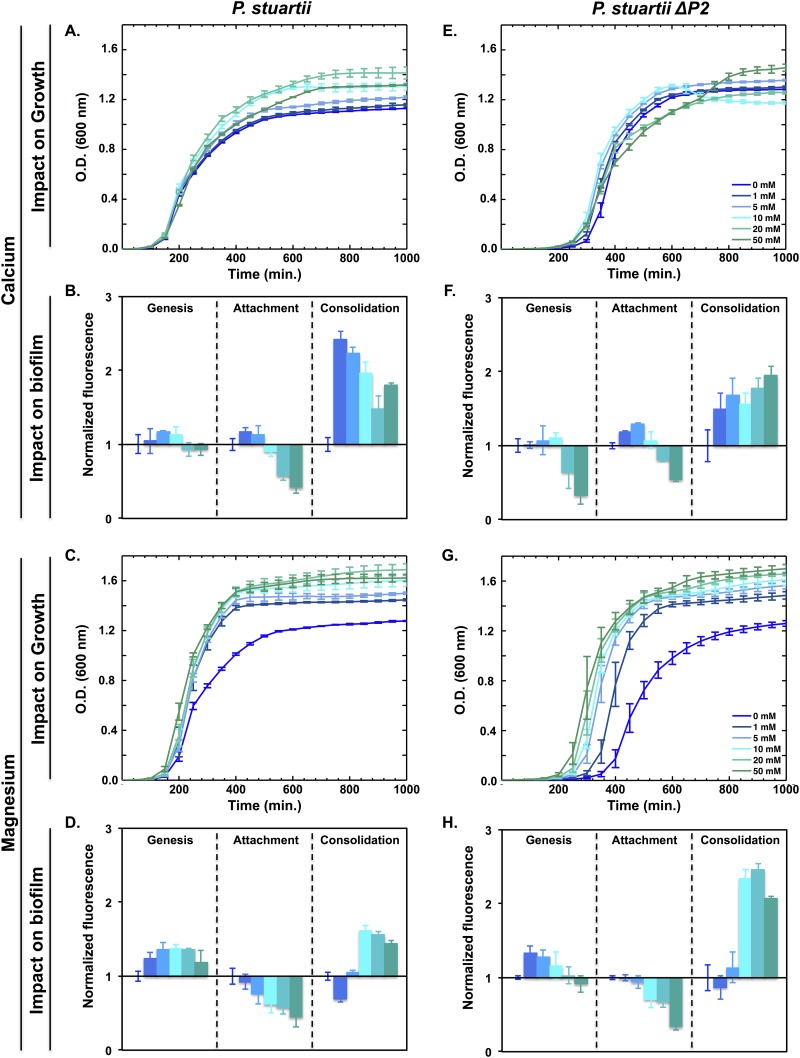
Resistance of *P*. *stuartii* and *P*. *stuartii*-ΔP2 cells to high concentrations of calcium and magnesium. Effect of calcium (A and E) and magnesium (C and G) on bacterial growth, as judged from the O.D. of cultures at 600 nm. The impact on biofilm genesis, attachment and consolidation was evaluated by adding calcium (B and F) or magnesium (D and H) during the lag, stationary and preformed biofilm phases, respectively. Biofilm formation was quantified 24 h later, by PrestoBlue staining. Fluorescence values were normalized with respect to the control wells buffered at pH7 and devoid of urea.

Calcium and magnesium are the most abundant divalent-cations in the urine. Therefore, their effect on the growth and fitness of *P*. *stuartii* cells was investigated, at the planktonic and biofilm levels. Upon addition of 50 mM of calcium and magnesium into the growth medium, *P*. *stuartii* growth level increased by 17 and 25% respectively (Fig 5A and 5C). Addition of calcium had an inhibitory effect on biofilm genesis as well and on cell attachment onto the surface, but triggered the consolidation of preformed-biofilms (Fig 5B) and a drastic reorganisation of their supracellular structure. Micrographs indeed reveal that *P*. *stuartii* biofilms change shape, forming compact assemblies of tightly aggregated cells (S2 Fig), upon exposure to high calcium concentration. Magnesium also inhibited cell attachment to surfaces, but was beneficial to biofilm genesis and consolidation (Fig 5D) and did not affect the shape of pre-formed biofilms, even at the highest concentrations tested.

Deletion of Omp-Pst2 reproduced or enhanced the impact of the two divalent cations on *P*. *stuartii* ΔP2 cells, planktonic and biofilm alike. The beneficial effect of calcium on planktonic growth was preserved, and likewise for its inhibitory effect on biofilm genesis and cell attachment onto the surface (Fig 5B and 5F). Pre-formed biofilms of *P*. *stuartii ΔP2* also displayed more sensitivity to high calcium concentrations, as exposed by the observation of more compact cell-assemblies characterized by a higher mortality level (S2 Fig), in epifluorescence micrographs. The beneficial effect of magnesium on planktonic growth was also preserved in *P*. *stuartii ΔP2* cells, while that on biofilm consolidation was further potentiated (Fig 5H).

## Discussion

*P*. *stuartii* is a biofilm-forming pathogen (Fig 1B), mainly involved in urinary tract infections in elderly patients [11,15]. It is occasionally implicated in others types of infections [47], including endocardisis [20], pericarditis [21], peritonitis [22] and meningitis [23]. Notwithstanding that the prevalence of infections is increasing alarmingly, knowledge regarding the species and its biofilms remains scarce.

Here, we aimed at studying *P*. *stuartii* growth and biofilm formation in conditions that mimic its most common habitat in humans, *i*.*e*. the urinary tract. Our study demonstrated the ability of the microbe to form biofilms in a variety of insulting conditions. Furthermore, it shed light on the highly gregarious behaviour of *P*. *stuartii* planktonic cells, which associate from the early stages of growth through cell-to-cell contact, forming floating community of cells prior to formation of canonical—per say, adherent—biofilms (Fig 2 and S1 Movie). Cell-to-cell contact in the planktonic state could provide a means to maximize cell density, inter-communication and resistance to environmental cues, before formation of the mature biofilm. We observed that the floating communities of cells could attach altogether to surfaces, allowing biofilms to start from a critical mass.

Planktonic and biofilm cells of *P*. *stuartii* adapt well to changes in pH, in the pH 6 to 9 range. Alkaline pH furthermore activates *P*. *stuartii* biofilm genesis and attachment to surfaces (Fig 4A-4D). This fitness suggests the existence of periplasmic pH regulators, which would support the swift adaptation of *P*. *stuartii* to pH variation. *P*. *stuartii ΔP2*, plagued by delayed growth, displays a clear dependence of the lag time on pH, suggesting that Omp-Pst2 contributes to the ability of the parental strain to immediately grow regardless of pH (Fig 4C and 4G). Thus Omp-Pst2 could play a role in regulating/buffering periplasmic pH, thereby supporting the adaptation of wild-type *P*. *stuartii* to changes in environmental pH. In the absence of this presumed regulator, *P*. *stuartii ΔP2* cells would take longer to adapt to increasing pH, hence explaining their retarded growth (+300 min at pH 9, compared to pH 7, Fig 4G).

The urease activity of *P*. *stuartii ATCC 29914* was uncovered by its ability to grow normally in mediums containing up to 500 mM urea. The species furthermore survives (15%) in 1 M urea (Fig 4A) demonstrating that it would be able to grow in the urinary tract. *P*. *stuartii ΔP2* is nevertheless more sensitive to urea, displaying an increased lag time at 500 mM urea (+ 250 min) and showing no growth at 1M urea (Fig 4E). Accordingly, biofilm genesis, attachment and consolidation by this strain are affected by urea in a dose-dependent manner (Fig 4F). This mechanism of toxicity is not detectable in the parental strain. It appears unlikely that Omp-Pst2 absence would effect in increasing urea concentration in the periplasm. Rather, Omp-Pst2 is involved in a process downstream of the penetration of urea into cells, such as efflux of urea or of its hydrolytic products. We see at least three possible explanations for the reduced resistance to urea of the *P*. *stuartii* ΔP2 strain. (i) Omp-Pst2 could contribute to the efflux of urea from the periplasm, hence reducing the actual periplasmic (and thus cytoplasmic) concentration of urea. Given the large excess present in the surrounding growth medium, it nevertheless appears unlikely that this efflux activity would significantly affect the periplasmic concentration in urea. (ii) Omp-Pst2 could facilitate the influx of anions neutralizing the periplasmic ammonium generated by urea degradation—but again, given the strong cation-selectivity of Omp-Pst2, however, this hypothesis also appears unlikely. (iii) Omp-Pst2 could be involved in the direct efflux of periplasmic ammonium and in limiting its repenetration into the periplasm; this is the only hypothesis that fits the prior knowledge on Omp-Pst2. Indeed, MD simulations have suggested that Omp-Pst2 could be involved in the facilitated transport of cations from the periplasm to the extracellular side of the OM [42]. They furthermore highlighted that Omp-Pst2 channel would respond by gating to a massive transit of cations from the external medium to the periplasm [42]. Thus, we favour the hypothesis that Omp-Pst2 alleviates the toxic effects of urea on *P*. *stuartii* cells by mitigating the toxic accumulation of ammonium in their periplasm. Of note, rough calculations indicate that accumulation of 10 μM ammonium would raise the periplasmic pH from 7 to 9. Thus, the delayed growth displayed by *P*. *stuartii ΔP2* cells in the presence of 500 mM urea could result from increased periplasmic pH, due to reduced efflux of ammonium. More generally, Omp-Pst2 could act on periplasmic pH by regulating the cationic content of the periplasm; this hypothesis will have to be confirmed by electrophysiology.

Elevated calcium and magnesium consolidate preformed *P*. *stuartii* biofilms, activate planktonic cell growth (Fig 5A and 5C), and reduce cell attachment onto surfaces (Fig 5B and 5D). Calcium at 50 mM induces changes in the macroscopic appearance of biofilms, leading to their compaction (S2 Fig), whereas magnesium slightly activates their genesis. These effects are further exaggerated in *P*. *stuartii ΔP2* (Fig 5F and 5H), again supporting that the absence of Omp-Pst2 could lead to accumulation of cations in the periplasm, aggravating the effect of these on cellular metabolism.

Altogether, our results suggest that *P*. *stuartii* exploits sociability as a means to foster cellular growth and resist to environmental stress, before formation of a canonical—per say, surface-attached—biofilm. Our work also points out that Omp-Pst2 plays a crucial role in the early stages of *P*. *stuartii* growth (Fig 1A). Data show that Omp-Pst2 is involved in pH regulation (Fig 4G) and could be in charge of ammonium clearance/neutralization from/in the periplasm, a hypothesis supported by electrophysiology data [40] and more recently molecular dynamics simulations on the porin [42]. Our data add to the growing body of evidence that suggests an implication of OM components in biofilm formation and regulation [48,49]. Further studies are needed to understand how *P*. *stuartii* cells are riveted to one another in floating communities of planktonic cells, and how this phenotype influences the ability of the species to form biofilms.

## Supporting information

S1 Movie *P*. *stuartii* forms floating communities of cells. Bacteria were grown to an O.D. of 0.5 at 600 nm and imaged by conventional microscopy without washing. The movie shows a Z-scan acquisition of the whole well (bottom-to-top). Planktonic cells assemble into a floating community, wherein cells appear to be attached to one another and are presumably in close contact.
(M4V)

S1 Fig Impact of urea and pH impact on biofilm genesis. Effect of urea (B-C and H-I) and pH (D-F and J-L) on the genesis of *P*. *stuartii* (A-F) and *P*. *stuartii ΔP2* (G-L) biofilms. Cells were subjected to environmental stresses for 24 h and bacteria adherent to the well surface were imaged after discard of planktonic cells by PBS washes. Live and dead cells were stained with SYTO9 Green and propidium iodide, respectively. Scale bar: 100 μm.
(TIF)

S2 Fig Impact of urea, pH and divalent cations on consolidation of pre-formed biofilms. Effect of urea (B, H), calcium (E, K), magnesium (F, L) and pH (C- D, I-J) on the consolidation of *P*. *stuartii* (A-F) and *P*. *stuartii ΔP2* (G-L) biofilms. Cells were subjected to environmental stresses for 24 h and bacteria adherent to the well surface were imaged after discard of planktonic cells by PBS washes. Live and dead cells were stained with SYTO9 Green and propidium iodide, respectively. Scale bar: 100 μm.
(TIF)
