# Supplementary figures and images for: Providencia stuartii form biofilms and floating communities of cells that display high resistance to environmental insults
Source: PLoS One. 2017 Mar 23;12(3):e0174213. doi: 10.1371/journal.pone.0174213 (PMC5363852; doi:10.1371/journal.pone.0174213)

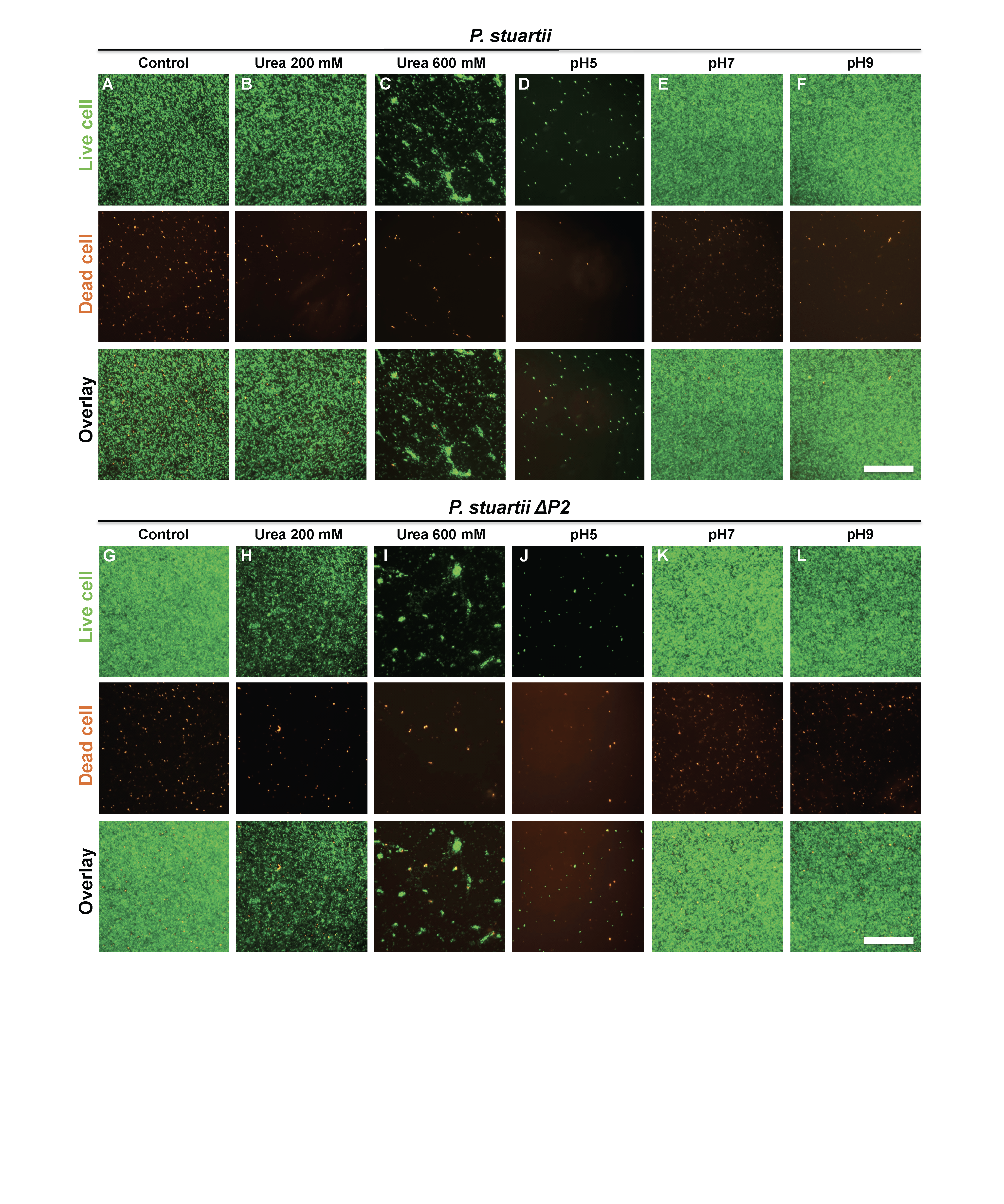

Supplement: S1 Fig — Effect of urea (B-C and H-I) and pH (D-F and J-L) on the genesis of P. stuartii (A-F) and P. stuartii ΔP2 (G-L) biofilms. Cells were subjected to environmental stresses for 24 h and bacteria adherent to the well surface were imaged after discard of planktonic cells by PBS washes. Live and dead cells were stained with SYTO9 Green and propidium iodide, respectively. Scale bar: 100 μm. (TIF) [file pone.0174213.s002.tif]

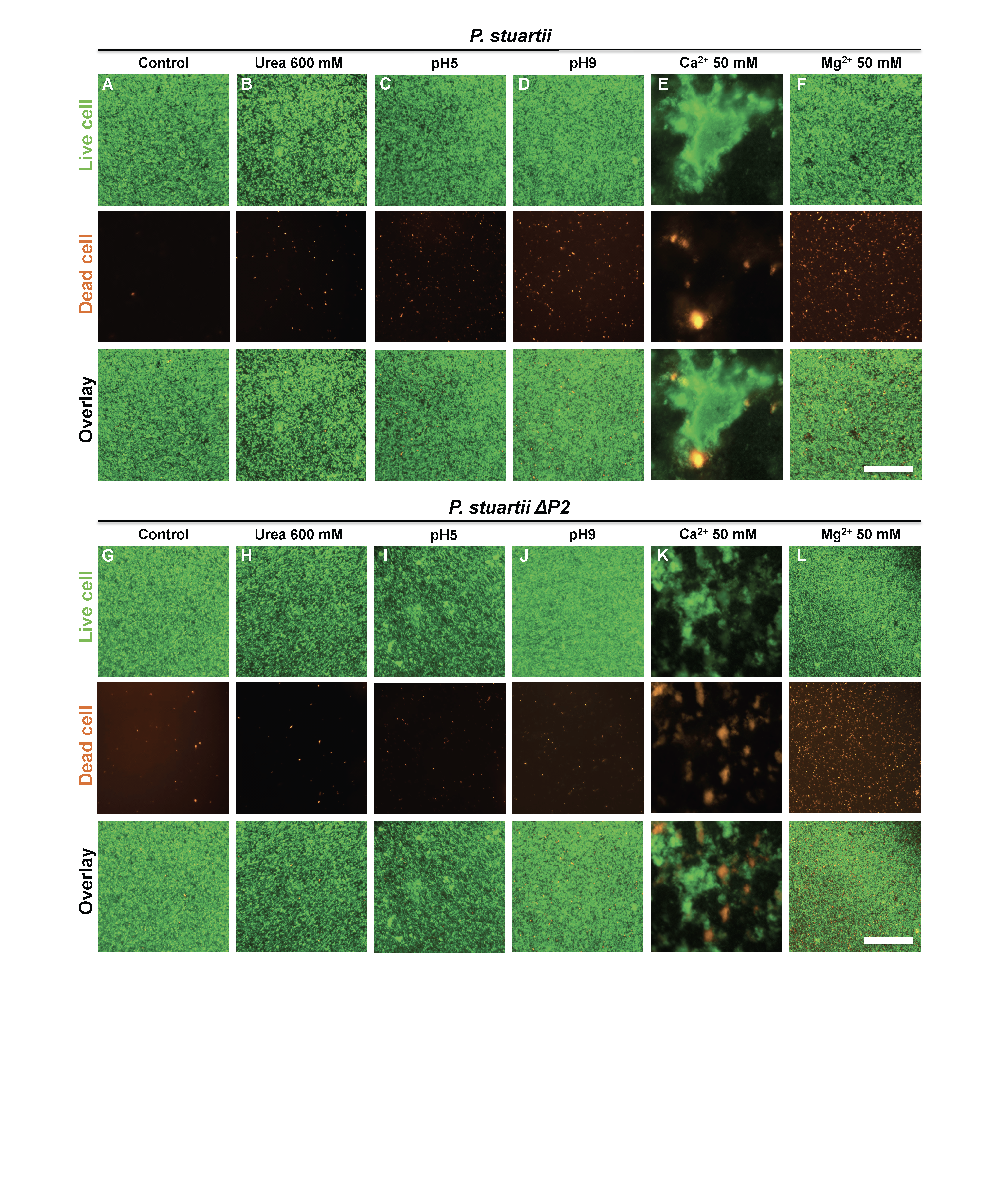

Supplement: S2 Fig — Effect of urea (B, H), calcium (E, K), magnesium (F, L) and pH (C- D, I-J) on the consolidation of P. stuartii (A-F) and P. stuartii ΔP2 (G-L) biofilms. Cells were subjected to environmental stresses for 24 h and bacteria adherent to the well surface were imaged after discard of planktonic cells by PBS washes. Live and dead cells were stained with SYTO9 Green and propidium iodide, respectively. Scale bar: 100 μm. (TIF) [file pone.0174213.s003.tif]
